# Prognostic value of serum thymidine kinase, tissue polypeptide antigen and neuron specific enolase in patients with small cell lung cancer.

**DOI:** 10.1038/bjc.1991.309

**Published:** 1991-08

**Authors:** A. van der Gaast, W. L. van Putten, R. Oosterom, M. Cozijnsen, R. Hoekstra, T. A. Splinter

**Affiliations:** Department of Medical Oncology, University Hospital Rotterdam-Dijkzigt, The Netherlands.

## Abstract

In a group of seventy patients with small cell lung cancer the prognostic value of serum tumour markers was determined. Thymidine kinase (TK), tissue polypeptide antigen (TPA) and lactate dehydrogenase (LDH) but not neuron specific enolase (NSE) correlated significantly with survival. Since all markers were strongly interrelated with each other and with the extent of disease, the combined determination of TK, TPA and LDH or the combination of disease extent and a marker yielded no more prognostic information than a single measurement of one of these variables.


Br. J. Cancer (1991), 64, 369-372 © Macmillan Press Ltd., 1991

### Prognostic value of serum thymidine kinase, tissue polypeptide antigen and neuron specific enolase in patients with small cell lung cancer

A van der Gaast[1], W.L.J. van Putten[2], R. Oosterom[3], M. Cozijnsen[1], R. Hoekstra[1] & T.A.W. Splinter[1]

*Department of [1]Medical Oncology, University Hospital Rotterdam-Dijkzigt, The Netherlands; Departments of [2]Biostatistics and [3]Clinical Chemistry, The Dr Daniel den Hoed Cancer Center, The Netherlands.*

**Summary** In a group of seventy patients with small cell lung cancer the prognostic value of serum tumour markers was determined. Thymidine kinase (TK), tissue polypeptide antigen (TPA) and lactate dehydrogenase (LDH) but not neuron specific enolase (NSE) correlated significantly with survival. Since all markers were strongly interrelated with each other and with the extent of disease, the combined determination of TK, TPA and LDH or the combination of disease extent and a marker yielded no more prognostic information than a single measurement of one of these variables.

Well established prognostic factors in small cell lung cancer (SCLC) are performance status (PS) and extent of disease (Maurer *et al.*, 1981). Recently several groups have introduced biochemical data such as liver function tests, LDH, alkaline phosphatase, albumin haemoglobin, white blood cell count and bicarbonate to delineate prognostic subgroups in combination with PS and extent of disease or even as a replacement of extent (Souhami *et al.*, 1985, Cerny *et al.*, 1987). The prognostic significance of such biochemical data is probably either related to the tumourload and/or the site of metastases. The disadvantage of such an approach is that these data are an indirect reflection of the tumour. It seems more logical to investigate tumour derived products, which may give a direct estimation of the total body tumourload in a more refined way than the usual distinction between limited (LD) and extensive disease (ED). Several studies have been published on the prognostic value of tumour markers, but only few have compared the relative value of different prognostic factor (Deuss *et al.*, 1987; Buccheri *et al.*, 1987; Carney *et al.*, 1982).

The purpose of this study was to determine the prognostic value of three tumour markers for response and survival in comparison with LDH, PS and extent of disease in a group of uniformly staged and treated patients.

### Materials and methods

### *Patients*

Pretreatment blood samples were obtained from 70 consecutive, previously untreated patients with a histological diagnosis of undifferentiated small cell carcinoma of the lung. Minimal staging procedures for all patients included physical examination, chest X-ray, computed tomography scan or ultrasound of the upper abdomen, bone scan and unilateral bone-marrow biopsy. Limited disease (LD) was defined as disease confined to one hemithorax including ipsilateral hilar, mediastinal lymph nodes, and supraclavicular lymph nodes and extensive disease (ED) as disease spread beyond the hemithorax including extension to the chest wall or to the contralateral lung. In 61 patients, chemotherapy consisted of doxorubicin 45 mg m$^{-2}$ intravenously (IV) on day 1, cyclophosphamide 1000 mg m$^{-2}$ IV on day 1, and etoposide 100 mg m$^{-2}$ IV on days 1, 3 and 5, repeated every 3 weeks for at least five cycles provided no progression occurred. The nine remaining patients were treated with various chemotherapy regimens in most instances including a combination of carboplatin or cisplatin and etoposide or ifosfamide. Disease response was evaluated according to standard WHO criteria after every two or five courses. Patients with rapidly progressive disease after one course of chemotherapy were documented as progressive disease. Survival was recorded from the start of treatment to death.

### *Marker assessment*

NSE was measured with the Pharmacia NSE RIA test (Pharmacia AB, Uppsala, Sweden) as is described previously (Cooper *et al.*, 1985). Serum concentrations above 12.5 ng ml$^{-1}$ were considered to be pathological. Serum TK activity was determined by the method of Gronowitz (Gronowitz *et al.*, 1984) using a commercial radioenzymatic assay (Sangtec Medical Co, Bromma, Sweden) with $^{125}$iodo deoxyuridine as a substrate. TK levels above 5 U ml$^{-1}$ were considered as abnormal. TPA measurements were performed using a commercial kit (Prolifigen RIA Sangtec Medical Co, Bromma, Sweden). The cut-off level for normal TPA values was 100 U l$^{-1}$.

### *Statistical methods*

The variables NSE, TK, TPA and LDH were log transformed before calculation of correlation coefficients and Cox regression analysis. Extent of disease was classified and coded as 1 = limited disease and 2 = extensive disease. Response was classified and coded as 1 = complete response, 2 = partial response and 3 = no response. The strength of the association between the markers, extent of disease, performance status, response and survival was expressed using linear correlation coefficients. However, Cox's regression analysis was applied to determine the relative importance of the prognostic factors on survival.

### Results

Clinical characteristics are shown in Table I. Follow-up at time of analysis was completed for all patients but one, who was still alive 140 weeks after start of treatment. Pretreatment levels of serum NSE, TK and TPA could be determined in 69, 70 and 61 patients respectively. The percentage of patients with elevated markers in relation to the extent of disease is shown in Table II. Median NSE for patients with LD was 23 ng ml$^{-1}$ (range 8-132), and 71 ng ml$^{-1}$ (range

Correspondence: A. van der Gaast, Department of Medical Oncology, University Hospital Rotterdam-Dijkzigt, Dr. Molewaterplein 40, 3015 GD Rotterdam, The Netherlands.
Received 6 July 1990; and in revised form 11 February 1991.

**Table I** Patient characteristics

| | |
|---|---|
| No. patients | 70 |
| Median age (years) | 63 (range 36–75) |
| Sex | |
| male | 54 (77%0 |
| female | 16 (13%) |
| Median performance status (Karnofsky) | 70 (range 30–100) |
| Limited disease | 29 (41%) |
| Extensive disease | 41 (59%) |
| Response | |
| complete response | 27 (39%) |
| partial response | 30 (43%) |
| stable disease | 5 ( 7%) |
| progression | 3 ( 4%) |
| non-evaluable | 5 ( 7%) |
| Median survival all patients (weeks) | 40 (range 0–143) |
| median survival limited disease | 56 (range 13–140) |
| median survival extensive disease | 30 (range 0–143) |

**Table II** Percentage of patients with elevated markers in relation to the extent of disease

| | No. of patients and (%) | | |
|---|---|---|---|
| *Extent of disease* | *NSE* | *TK* | *TPA* |
| Limited disease | 26 (90) | 11 (38) | 9 (36) |
| Extensive disease | 38 (95) | 28 (68) | 27 (79) |

8–626) for patients with ED. Median TK and TPA for patients with LD were 5 U $l^{-1}$ (range 2–15) and 63 U $l^{-1}$ (range 17–311) respectively, and 17 U $l^{-1}$ (range 2–400) and 311 U $l^{-1}$ (range 24–7076) for patients with ED.

The correlation coefficients between the serum markers, LDH, Performance score according to the Karnofsky index, response and survival are shown in Table III. A significantly intermarker correlation was observed and also between the markers and serum LDH. An example of a scatter diagram of TPA and NSE is depicted in Figure 1. All markers as well as LDH correlated with the stage of disease. This correlation was even more pronounced when patients with extensive disease were subdivided into two groups: patients with metastases to only one organ site for example only liver metastases or only bone metastases (ED–1), and patients with metastases to more than one organ site (ED–2) (Table IV). In univariate analyses PS, disease extent, TK, TPA and LDH were all variables significantly correlated with survival. NSE on the contrary was not significantly correlated with survival (Table V). Since many of the above mentioned variables were interrelated, a multivariate analysis of the various factors has been performed in order to determine their relative prognostic importance. In the final model only TK remained as an significant prognostic factor. Addition of one of the other variables or extent of disease to a model with TK did not improve the fit of the model ($P > 0.10$). However due to the relative limited sample size and the observed standard error it could not be significantly shown that TK was a better prognostic factor than TPA, LDH or extent of disease.

**Table III** The correlation coefficients between the markers, LDH, according to performance score, response and survival

| | *Parameters* | | | | | | |
|---|---|---|---|---|---|---|---|
| | *NSE* | *TK* | *TPA* | *LDH* | *PS* | *Resp.* | *Surv.* |
| NSE | 1 | | | | | | |
| TK | 0.57 | | | | | | |
| TPA | 0.71 | 0.61 | 1 | | | | |
| LDH | 0.72 | 0.70 | 0.84 | 1 | | | |
| PS | – 0.42 | – 0.44 | – 0.51 | – 0.57 | 1 | | |
| Resp. | 0.31 | 0.58 | 0.49 | 0.57 | – 0.34 | 1 | |
| Surv. | – 0.13 | – 0.37 | – 0.37 | – 0.35 | 0.25 | – 0.62 | 1 |

Abbreviations: NSE = log (neuron specific enolase); TK = log (thymidine kinase); TPA = log (tissue polypeptide antigen); LDH = log (lactate dehydrogenase); PS = performance score according to Kar nofsky; Resp. = response; Surv. = survival; LD = limited disease; ED = extensive disease.

### Discussion

The evaluation of prognostic factors may serve different purposes such as choice of treatment, individual prognostication, comparison of different trials or trialarms and gathering knowledge about the heterogeneity and biological behaviour of the disease.

**Figure 1** Scatter diagram of NSE and TPA.

**Table IV** Correlation coefficients and 95% confidence intervals between TK, TPA, NSE, LDH and disease extent

| | *TK* | *TPA* | *NSE* | *LDH* |
|---|---|---|---|---|
| Extent | 0.59 (0.41–0.72) | 0.54 (0.33–0.70) | 0.44 (0.23–0.61) | 0.48 (0.26–0.65) |

Abbreviations: TK = log (thymidine kinase); TPA = log (tissue polypeptide antigen; NSE = log (neuron specific enolase); LDH = log (lactate dehydrogenase); Extent = stage of disease grouped as limited disease, extensive disease-1 and extensive disease-2 (see text).

**Table V** Results of univariate Cox's proportional hazards regression analyses

| *Variable* | *Regression coefficient* | *SE* | *P* |
|---|---|---|---|
| TK | 0.44 | 0.13 | 0.001 |
| NSE | 0.13 | 0.14 | 0.36 |
| TPA | 0.37 | 0.12 | 0.003 |
| LDH | 0.56 | 0.20 | 0.008 |
| Extent | 0.65 | 0.25 | 0.01 |

Abbreviations: TK = log (thymidine kinase); TPA = log (tissue polypeptide antigen); NSE = log (neuron specific enolase); LDH = log (lactate dehydrogenase); Extent grouped as limited disease and extensive disease.

**Figure 2** Kaplan-Meier survival curve for different groups of patients based on their TK level. 1 = TK ⩽ 6 U ml; 2 = 6 U ml < TK ⩽ 18 U ml; 3 = TK > 18 U ml.

The prognosis is dependent on the combined effects of prognostic factors and therapy, which therefore understandably are frequently linked. In case of chemotherapy the prognosis will be mainly determined by the total body tumourload, the fraction of chemotherapy-resistant cells, either predetermined or acquired, and the growth rate of the latter. Theoretically one or a combination of marker substances, which will reflect one of the above mentioned prognostic factors, would seem to be ideal. One of the prerequisites for such a marker would be that the amount, which is released or secreted per gram of tumour tissue, is independent of tumour heterogeneity or correlated with a prognostically important difference in biological behaviour.

In this study it was found that NSE, TK, TPA and LDH were significantly correlated with the extent of disease, with each other and with PS. TK, TPA and LDH but not NSE were also significantly correlated with survival. The former observations are in agreement with previous reports (Deuss *et al.*, 1987; Buccheri *et al.*, 1987; Gronowitz *et al.*, 1986). The latter observation is not supported by the studies of Akoun *et al.* (1985) and Jorgensen *et al.* (1988) who showed that NSE is a significant prognostic factor for survival. The combined lack of correlation between NSE and both response and survival suggests that treatment related factors may be involved, i.e. that in contrast to the studies of Akoun *et al.* (1985) and Jorgenson *et al.* (1988) in our study a population of tumour cells is killed, which is correlated less with the serum NSE level. Approximately 85% of the patients in our study received a three-drug regimen consisting of cyclophosphamide, doxorubicin and etoposide. In the Danish study (Jorgenson *et al.*, 1988) the patients received three different regimens, each of which contained at least six different drugs such as CCNU, cyclophosphamide, vincristine, etoposide, doxorubicin, cisplatin, vindesine and hexamethylmelamine. In the French study (Akoun *et al.*, 1985) patients received four different drugs (cyclophosphamide, doxorubicin, vincristine and methotrexate). Although very different to prove it may be well that especially in the Danish study the six drugs killed a somewhat different population of tumour cells than the three drugs in our study. This may explain why serum NSE as a reflection of the tumourload is correlated with prognosis in one study and not the other.

TK, TPA, LDH and extent of disease all had significant prognostic capacity in the univariate analysis, however in the multivariate analysis it was shown that the combination of extent of disease and TK, TPA or LDH yielded no more prognostic information than the measurement of one of these parameters. Even a combination of the various markers didn't improve upon the significance of a single marker. This latter observation may suggest that all these markers are picking out the same cells or bear the same relationship with the total tumourload.

Besides the fact that TK seems to be a good indicator of the total tumour mass it could have been assumed that there might be a relation between TK and the proliferative activity of the tumours of patients with small cell lung cancer since TK is involved in the process of DNA synthesis. Clinical evidence for the relation of TK and growth rate has been provided by the study of Greengard *et al.* (1985) in which it has been observed that TK concentrations of biopsy samples bear a quantitative inverse correlation to the volume doubling time of lung neoplasms as determined by tumour diameter measurements from sequential chest X-rays. However, if TK in SCLC relates with tumour growth rate this would most probably result in different responses to treatment for tumours based on their TK levels resulting in survival curves with different slopes (Shackney *et al.*, 1978). Survival curves calculated by the Kaplan-Meier method for different groups of patients based on their TK levels are depicted in Figure 2. Since the slopes of both depicted survival curves are identical this suggests that the prognostic capacity of TK is most probably entirely tumourload related.

In conclusion, in this study TK, TPA, LDH and extent of disease, but not NSE, proved to be valuable prognostic factors for prediction of survival. The prognostic significance of all these variables seems to be entirely tumourload related. Further studies including more patients are warranted to evaluate whether a single measurement of a marker can replace a crude distinction between LD and ED determined through a complicated staging procedure.

### References

AKOUN, G.M., SCARNA, H.M., MILLERON, B.J., BENICHOU, M.P. & HERMAN, D.P. (1985). Serum neuron-specific enolase: a marker for disease extent and response to therapy for small cell lung cancer. *Chest*, **87**, 39.

BUCCHERI, G.F., FERRIGNO, D., SARTORIS, A.M., VIOLANTE, B., VOLA, F. & CURCIO, A. (1987). Tumor markers in bronchogenic carcinoma. *Cancer*, **60**, 42.

CARNEY, D.N., IDHE, D.C., COHEN, M.H. & 4 others (1982). Serum neuron-specific enolase: a marker for disease extent and response to therapy of small-cell lung cancer. *Lancet*, **i**, 583.

CERNY, T., BLAIR, V., ANDERSON, H., BRAMWELL, V. & THATCHER, N. (1987). Pretreatment prognostic factors and scoring system in 407 small-cell lung cancer patients. *Int. J. Cancer*, **39**, 146.

COOPER, E.H., SPLINTER, T.A.W., BROWN, D.A., MUERS, M.F., PEAKE, M.D. & PEARSON, S.C. (1985). Evaluation of a radioimmunoassay for neuron-specific enolase in small cell lung cancer. *Br. J. Cancer*, **52**, 333.

DEUB, U., ALLOLIO, B., KAULEN, D., WINKELMANN, W., ARNOLD, C. & PACK, I. (1987). Serum-thymidinkinase als tumormarker beim bronchialkarzinom: stadienabhangigkeit und verlaufskontrollen. *Tumor Diagnostik & Therapie*, **8**, 69.

GREENGARD, O., HEAD, J.F., GOLDBERG, S.L. & KIRSCHNER, P.A. (1985). Biochemical measure of the volume doubling time of human pulmonary neoplasms. *Cancer*, **55**, 1530.

GRONOWITZ, J.S., KALLANDER, C.F.R., DIDERHOLM, H., HAGBERG, H. & PETTERSSON, U. (1984). Application of an in vitro assay for serum thymidine kinase: results on viral disease and malignancies in humans. *Int. J. Cancer*, **33**, 5.

GRONOWITZ, J.S., STEINHOLTZ, L., KALLANDER, C.F.R., HAGBERG, H. & BERGH, J. (1986). Serum deoxythymidine kinase in small cell carcinoma of the lung. *Cancer*, **58**, 111.

JORGENSEN, L.G.M., OSTERLIND, K., HANSEN, H.H. & COOPER, E.H. (1988). The prognostic influence of serum neuron specific enolase in small cell lung cancer. *Br. J. Cancer*, **58**, 805.

MAURER, L.H. & PAJAK, T.J. (1981). Prognostic factors in small cell carcinoma of the lung: a cancer and leukemia group B study. *Cancer Treat. Rep.*, **65**, 767.

REEVE, J.G., STEWART, J., WATSON, J.V., WULFRANK, D., TWENTYMAN, P.R. & BLEEHEN, N.M. (1986). Neuron specific enolase expression in carcinoma of the lung. *Br. J. Cancer*, **53**, 519.

SHACKNEY, S.E., McCORMACK, G.W. & CUCHURAL, G.J. (1978). Growth rate patterns of solid tumors and their relation to responsiveness to therapy. *Ann. Intern. Med.*, **89**, 107.

SOUHAMI, R.L., BRADBURY, I., GEDDES, D.M., SPIRO, S.G., HARPER, P.G. & TOBIAS, J.S. (1985). Prognostic significance of laboratory parameters measured at diagnosis in small cell carcinoma of the lung. *Cancer Res.*, **45**, 2878.


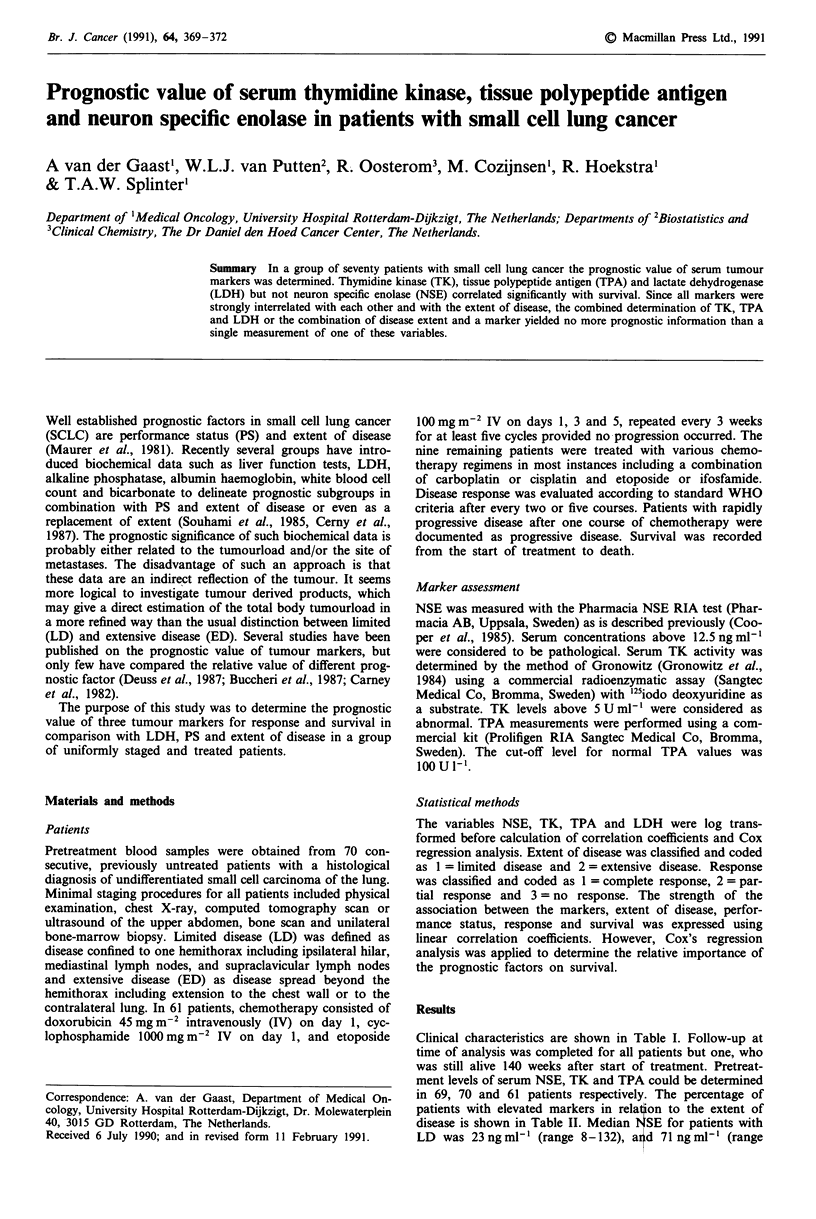

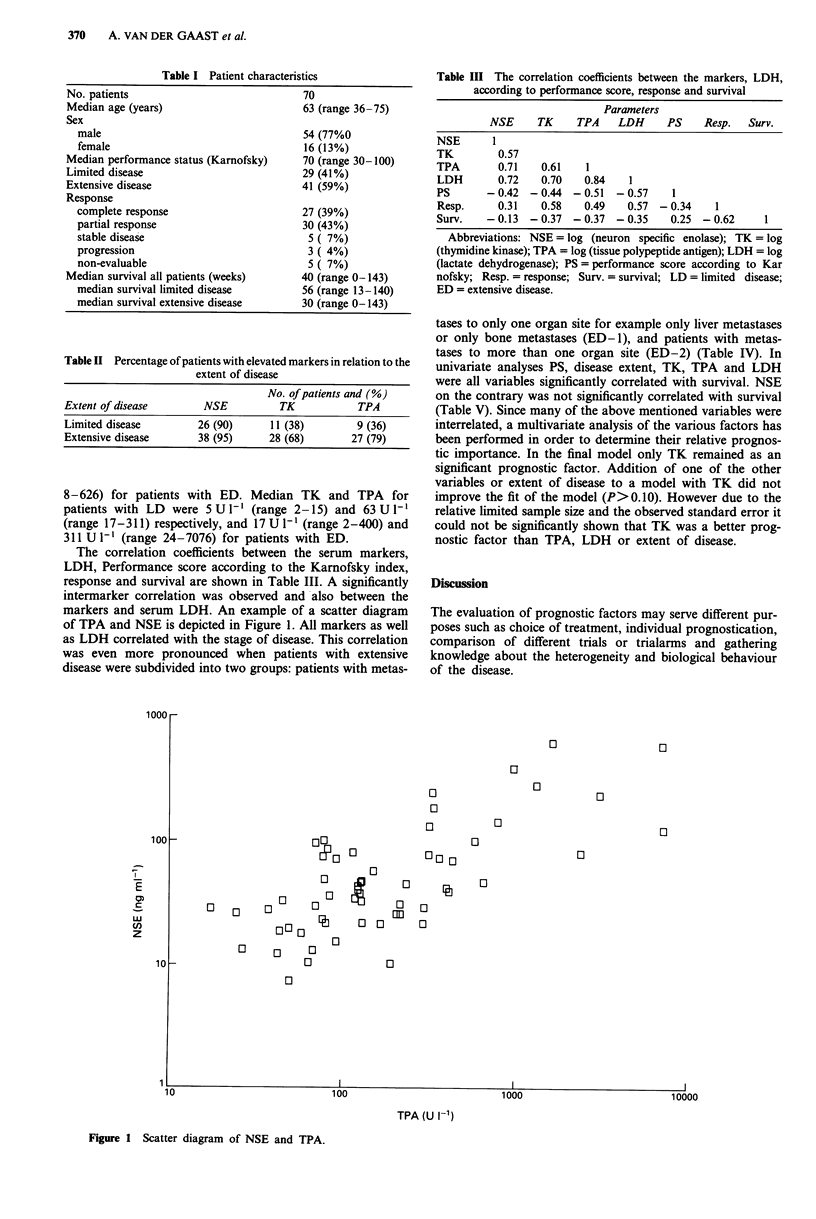

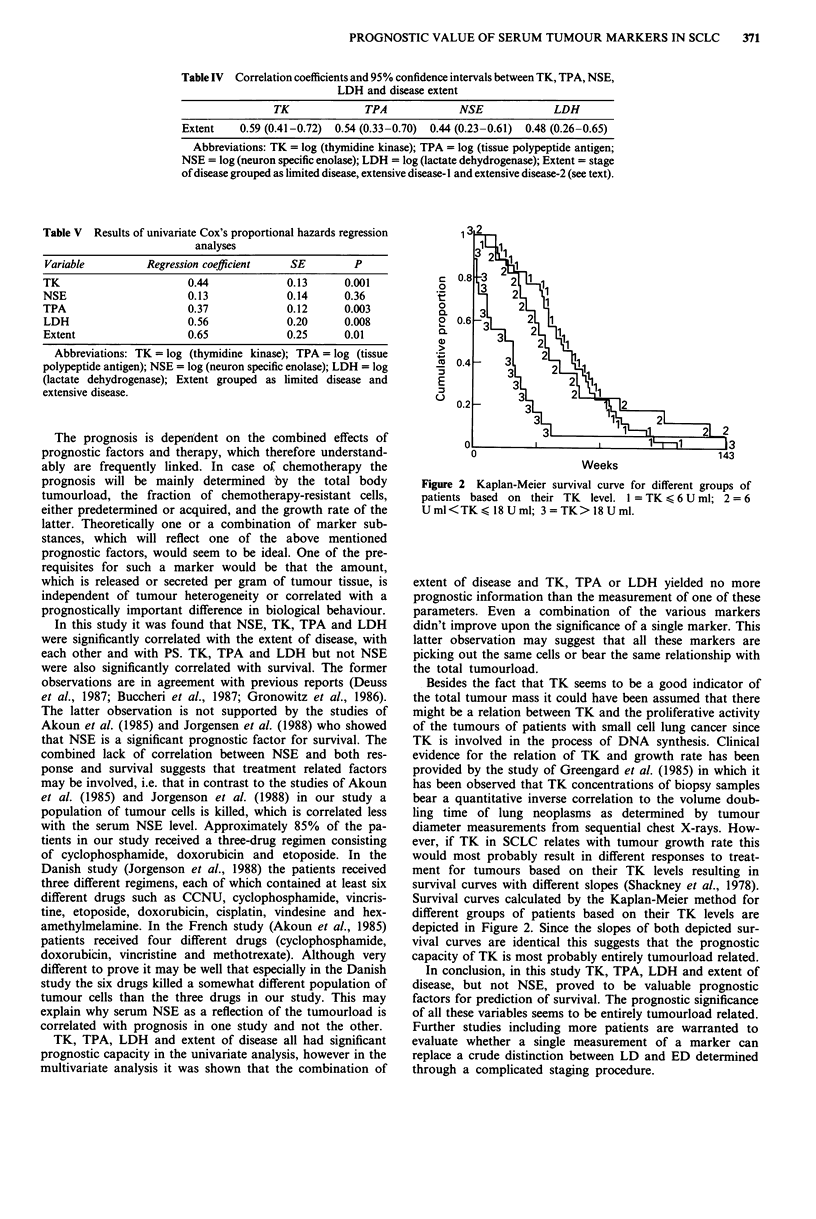

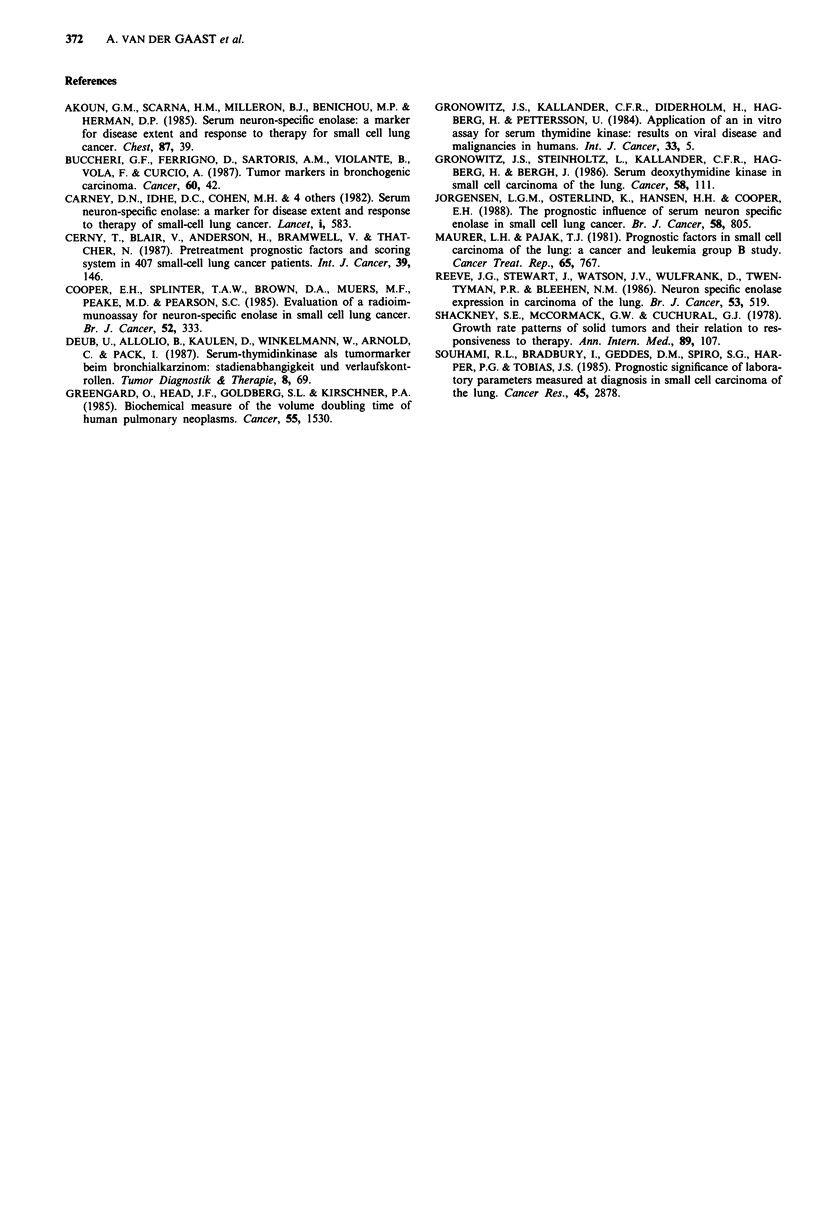


## References

[OCR_00491] Akoun G. M., Scarna H. M., Milleron B. J., Bénichou M. P., Herman D. P. (1985). Serum neuron-specific enolase. A marker for disease extent and response to therapy for small-cell lung cancer.. Chest.

[OCR_00497] Buccheri G. F., Ferrigno D., Sartoris A. M., Violante B., Vola F., Curcio A. (1987). Tumor markers in bronchogenic carcinoma. Superiority of tissue polypeptide antigen to carcinoembryonic antigen and carbohydrate antigenic determinant 19-9.. Cancer.

[OCR_00502] Carney D. N., Marangos P. J., Ihde D. C., Bunn P. A., Cohen M. H., Minna J. D., Gazdar A. F. (1982). Serum neuron-specific enolase: a marker for disease extent and response to therapy of small-cell lung cancer.. Lancet.

[OCR_00509] Cerny T., Blair V., Anderson H., Bramwell V., Thatcher N. (1987). Pretreatment prognostic factors and scoring system in 407 small-cell lung cancer patients.. Int J Cancer.

[OCR_00513] Cooper E. H., Splinter T. A., Brown D. A., Muers M. F., Peake M. D., Pearson S. L. (1985). Evaluation of a radioimmunoassay for neuron specific enolase in small cell lung cancer.. Br J Cancer.

[OCR_00525] Greengard O., Head J. F., Goldberg S. L., Kirschner P. A. (1985). Biochemical measure of the volume doubling time of human pulmonary neoplasms.. Cancer.

[OCR_00532] Gronowitz J. S., Källander F. R., Diderholm H., Hagberg H., Pettersson U. (1984). Application of an in vitro assay for serum thymidine kinase: results on viral disease and malignancies in humans.. Int J Cancer.

[OCR_00538] Gronowitz J. S., Steinholtz L., Källander C. F., Hagberg H., Bergh J. (1986). Serum deoxythymidine kinase in small cell carcinoma of the lung. Relation to clinical features, prognosis, and other biochemical markers.. Cancer.

[OCR_00541] Jørgensen L. G., Osterlind K., Hansen H. H., Cooper E. H. (1988). The prognostic influence of serum neuron specific enolase in small cell lung cancer.. Br J Cancer.

[OCR_00546] Maurer L. H., Pajak T. F. (1981). Prognostic factors in small cell carcinoma of the lung: a cancer and leukemia group B study.. Cancer Treat Rep.

[OCR_00553] Reeve J. G., Stewart J., Watson J. V., Wulfrank D., Twentyman P. R., Bleehen N. M. (1986). Neuron specific enolase expression in carcinoma of the lung.. Br J Cancer.

[OCR_00556] Shackney S. E., McCormack G. W., Cuchural G. J. (1978). Growth rate patterns of solid tumors and their relation to responsiveness to therapy: an analytical review.. Ann Intern Med.

[OCR_00563] Souhami R. L., Bradbury I., Geddes D. M., Spiro S. G., Harper P. G., Tobias J. S. (1985). Prognostic significance of laboratory parameters measured at diagnosis in small cell carcinoma of the lung.. Cancer Res.

